# Child Psychiatrists’ Knowledge and Attitudes on Medication Reimbursement Schemes in Ireland

**DOI:** 10.1192/bjo.2025.10120

**Published:** 2025-06-20

**Authors:** Orlagh Deighan, Aisling MacManus, Fiona McNicholas

**Affiliations:** ^1^Irish College of Psychiatry, Dublin, Ireland; ^2^University College Dublin, Dublin, Ireland

## Abstract

**Aims:** The Long-Term Illness Scheme (LTI), funded by Ireland’s Health Service Executive (HSE), provides free prescriptions at the primary care level for 16 specified physical and mental health conditions. This scheme is non means tested and is facilitated under the Primary Care reimbursement service via the HSE. It was initiated in 1970 under the Health Care Act and was last revised in 1975. This survey aimed to assess Child and Adolescent Psychiatrists’ knowledge of the scheme, its usage, and the perceived barriers or enablers to its utilization.

**Methods:** A mixed-methods cross-sectional survey was conducted among Consultants and Higher Specialist Trainees in Child and Adolescent Psychiatry in Ireland (N=60) on an anonymous, opt-in basis.

**Results:** The findings revealed low levels of knowledge and utilization with a minority (41%) of respondents reported being somewhat aware of the scheme. 58% felt uninformed about the medications reimbursable. Qualitative analysis highlighted significant barriers, including restrictive and confusing inclusion criteria, administrative burdens, and time constraints in clinical practice.

**Conclusion:** These results underline the need for national training on the LTI scheme, a systematic review of inclusion criteria to align with international best practices, and streamlined administrative processes. Addressing these gaps could reduce barriers for clinicians and improve access to psychotropic medications for children and adolescents with mental illness. Such measures are crucial to enhancing equitable care and alleviating administrative strain on consultants, ultimately benefiting both clinicians and young patients in Ireland. Equipping psychiatrists with the necessary tools and knowledge is essential to effectively utilize health reimbursement schemes and advocate for better mental health care.

